# Application of Lineage Tracing in Central Nervous System Development and Regeneration

**DOI:** 10.1007/s12033-023-00769-0

**Published:** 2023-06-19

**Authors:** Hao Li, Yuan Zhuang, Bin Zhang, Xiaojian Xu, Baiyun Liu

**Affiliations:** 1 Department of Neurosurgery, Beijing Tian tan Hospital, Capital Medical University, Beijing, China; 2 Beijing Key Laboratory of Central Nervous System Injury, Department of Neurotrauma, Beijing Neurosurgical Institute, Capital Medical University, Beijing, China; 3 Center for Nerve Injury and Repair, Beijing Institute of Brain Disorders, Beijing, China; 4 China National Clinical Research Center for Neurological Diseases, Beijing, China; 5 Department of Intensive Care Unit, Beijing Tian tan Hospital, Capital Medical University, Beijing, China

**Keywords:** Biotechnology, Brain development, Central nervous system disease, Gene targeting, Lineage tracing, Neurogenesis, Regeneration

## Abstract

The central nervous system (CNS) is a complicated neural network. The origin and evolution of functional neurons and glia cells remain unclear, as do the cellular alterations that occur during the course of cerebral disease rehabilitation. Lineage tracing is a valuable method for tracing specific cells and achieving a better understanding of the CNS. Recently, various technological breakthroughs have been made in lineage tracing, such as the application of various combinations of fluorescent reporters and advances in barcode technology. The development of lineage tracing has given us a deeper understanding of the normal physiology of the CNS, especially the pathological processes. In this review, we summarize these advances of lineage tracing and their applications in CNS. We focus on the use of lineage tracing techniques to elucidate the process CNS development and especially the mechanism of injury repair. Deep understanding of the central nervous system will help us to use existing technologies to diagnose and treat diseases.

## Introduction

In mammals, the brain is the most complex organ, and the complexity of neural networks is a major hurdle towards understanding the brain, especially post-injury changes. Numerous classical notions of brain functioning have been superseded [1, 2]. The relative lack of understanding of the brain has severely hindered the development of brain science, as well as the diagnosis and treatment of neurological diseases. Elucidating the processes underlying neural network formation, and the pathological changes that occur after injury, have been major goals of neuroscientists for many years [3]. Tracing the fates of specific cells using appropriate methods can provide a better understanding of brain development, as well as physiological and pathological brain processes. For decades, lineage tracing has been used as an effective strategy to explore neural networks at the cellular level, as well as brain repair processes after injury at the single-cell level, using specific cell markers [4]. For in vivo cell fate studies, genetic lineage tracing represents a powerful approach for tracking and understanding cell lineages without the requirement for artificial manipulation in vitro.

In this review, we summarize recent developments in the application of lineage tracing technology to the study of the mammalian brain, with the aim of providing an integrated perspective on physiological and pathological brain processes. In particular, we focus on the cellular origins of these processes under healthy and injured conditions, as well as the transformations that occur in the latter. We anticipate that this review will lay a foundation for further brain research and inform clinical treatment.

## Development of the Lineage Tracing Technique

Lineage tracing, a technique pioneered by Charles Whitman for tracking the evolution of cells and their progeny, has undergone rapid development over the last few decades. In lineage tracing, specific cells are marked, and the transmission of these markers to their progeny provides information on the migration and differentiation of cells [5]. Different lineage tracing techniques have unique characteristics (Table 1). The first lineage tracing method was direct observation using time-lapse microscopy, but it is difficult to explore the long-term fates of specific cells in vivo using this technique [6]. To improve the resolution of lineage tracing, lipid-soluble carbocyanine dyes, such as octadecyl indocarbocyanines and oxacarbocyanine, and substrate-activated horseradish peroxidase and DNA/histones can be used to directly label cells and determine the fate of their progeny [7–10]. Although transplantation has also been extensively applied in many systems (especially the skin, blood and muscle), it is not ideal for mimicking endogenous processes [11].

**Table 1:** The advantages and disadvantages of different lineage tracing methods [4, 11, 27, 87]

Method	Advantage	Disadvantage
Direct observation	Fast; easy; noninvasive	Limited application
Labeling cells with dyes	Better visibility	Limited scalability and duration
Transfection or viral transduction	Heritable	Low efficiency; indiscriminate infection; for retroviruses: only dividing cells; spontaneously silence
Transplantation of Cells and tissues	Permanent; distinguishing	Low integration and scalability; Invasive
Genetic molecular dye	Dynamically lineage tracing; inducible; stable; accurate; high efficiency	Long operating cycle; toxic lack temporal lineage; cell group
Sc-RNA-seq	High resolution; accurate; lineage tree; spatiotemporal tracing; diversification	Expensive; conditional; complexity; immature

**Fig. 1:**
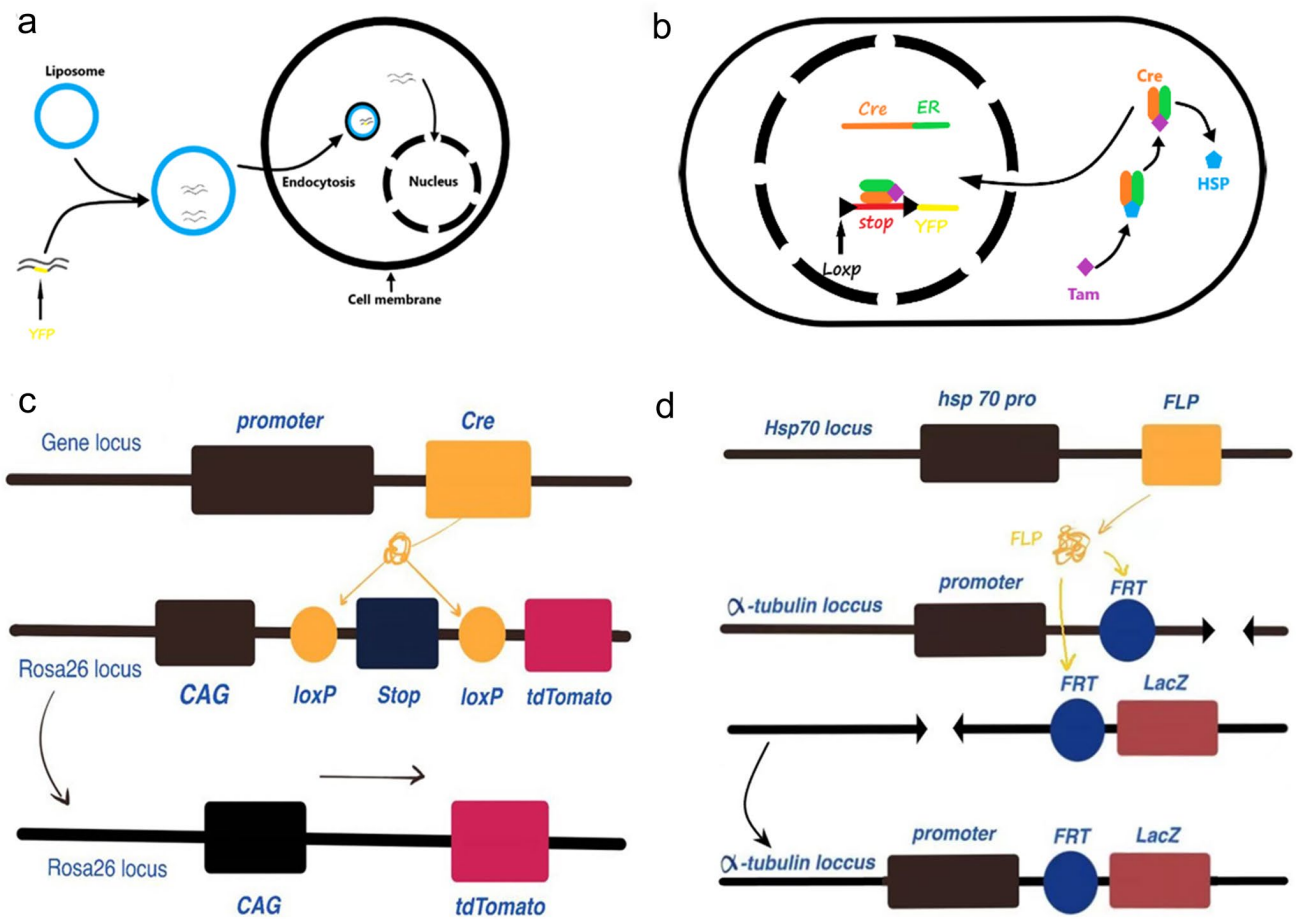
Schematic representation of transfection-mediated genetic recombination. **a** Transfection; **b** Site-specific recombination; **c** Cre-loxP system; **d** FLP-FRT system

With advances in molecular genetics, markers can now be expressed more rapidly and stably in cell lines via transfection or viral transduction [12, 13] (Fig. 1a). Adult hippocampal quiescent neural stem cells (NSCs) can serve as a target for lineage tracing and functional analyses involving recombinant adeno-associated virus serotype 4, and moreover, their activity can be manipulated [14]. Since the end of the twentieth century, genetic recombination has been widely used to label specific cell types in vivo. Fluorescently labeled gene sequences, such as cyclization recombination enzyme-locus of X-overP1 (Cre-lox P), flippase-flippase recognition target (FLP-FRT), D6 site-specific DNA recombinase-rox (Dre-rox) and Nigri-nox, have been inserted near target genes and detected in cell lines by fluorescence imaging [15]. At present, the most widely used site-specific gene recombination systems are Cre-lox P and FLP-FRT, which are derived from bacteriophages and *Saccharomyces cerevisiae*. The spatiotemporal activity of these systems can be regulated by a promoter from the human estrogen receptor gene [16–18] (Fig. 1b–d). To further improve our understanding of the division and origins of neurons and other cells via lineage tracing, a diverse range of recombinant enzymes have been applied. Neural progenitor cells and double markers have improved our understanding of neuronal origins, and lineage tracing has shed light on patterns of division [19, 20]. Cre/lox recombination has been used to create stochastic choice models of the expression of three or more fluorescent proteins. The connections and communication among brain cells can be understood by analyses of random expression [21].

Because of the high accuracy and sensitivity of sequencing technology, it has been widely applied for lineage tracing. Single-cell RNA-sequencing (scRNA-seq) has been favored because of its high resolution and ability to reveal heterogeneity among cell subpopulations [22]. Similar to traditional lineage tracing methods, sequencing methods can be classified as invasive or non-invasive. Although somatic mutations serving as non-invasive lineage markers can be detected in humans by the sequencing and reconstruction of lineage trees, it is difficult to achieve precise control with this method. Classical analytical and statistical methods have also been extended to single-cell technologies [23]. For example, combining scRNA-seq and brain registration, Pandey created a comprehensive map of the zebrafish habenula [24]. Similar to fluorescence lineage tracing, scientists have begun applying viruses and plasmids for barcoding. Although recent studies have combined scRNA-seq and clustered regularly interspaced short palindromic repeat-associated nuclease 9 (CRISPR-Cas9) editing systems to construct lineage trees and conduct neuroanatomy studies, myriad challenges remain [25, 26]. The application of barcoding to mammalian research has been limited [27]. Because Cre recombinases tend to excise rather than flip, barcode-based Cre-recombinase techniques gradually fail. The Poly-lox method and homing guide RNA (a novel CRISPR/Cas9 system) overcome the lack of diversity in barcodes and can be applied to the mammalian brain [28]. Bowling introduced CRISPR array repair lineage tracing to investigate liver hematopoietic stem cell clones in adult tissues, and obtained valuable insight into the application of barcodes to the study of adult mammals [29].

## Application of Lineage Tracing for Understanding Brain Development

**Fig. 2:**
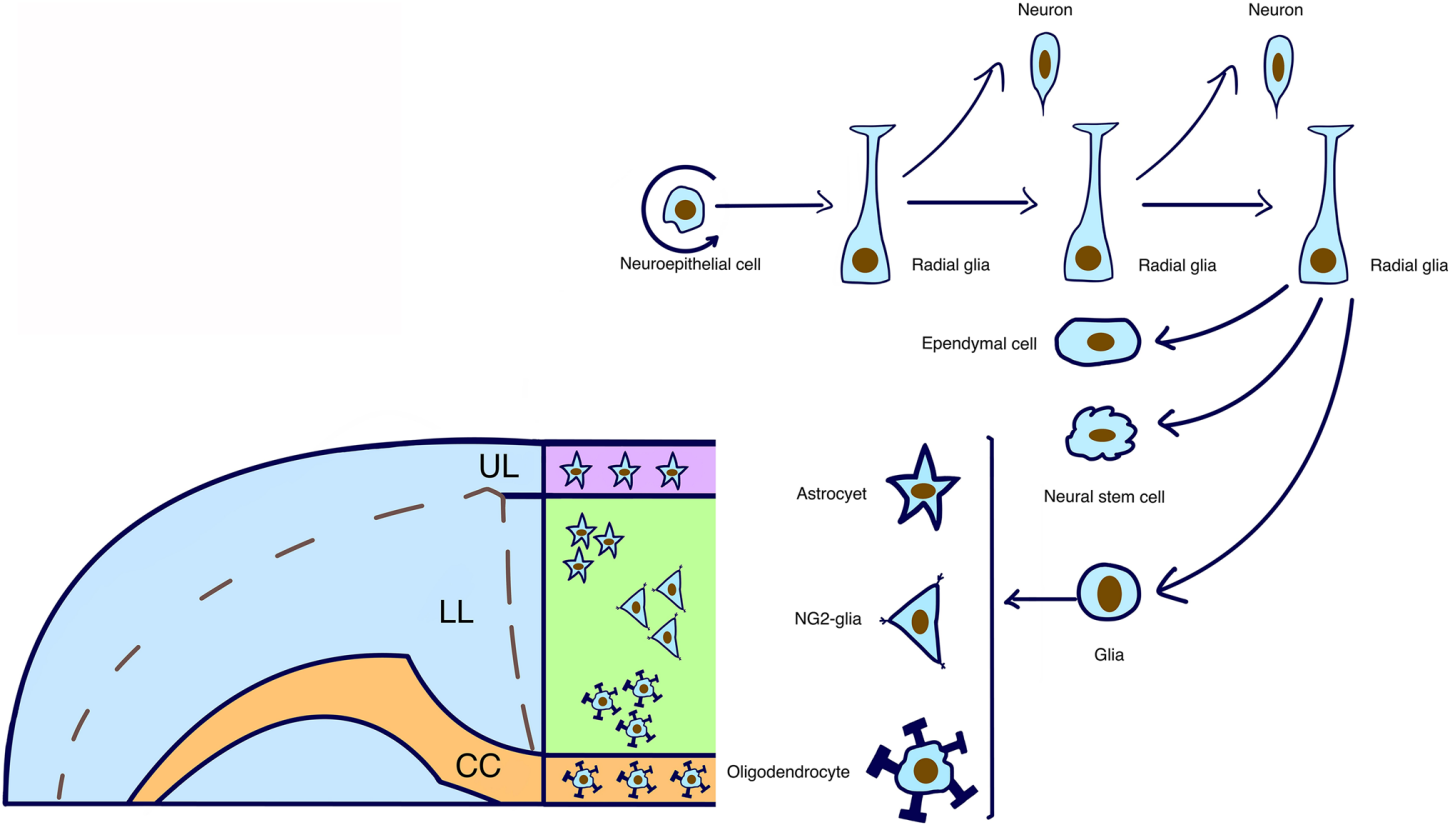
Cortical development and glial progeny arrangement in mouse. Several types of cortical progenitors and their modes of division towards different cells: neuroepithelial cells, radial glial cells, neurons, with their specific location in UL (upper cortical layers), CC (callous corpus), and LL (lower cortical layers) regions

**Fig. 3:**
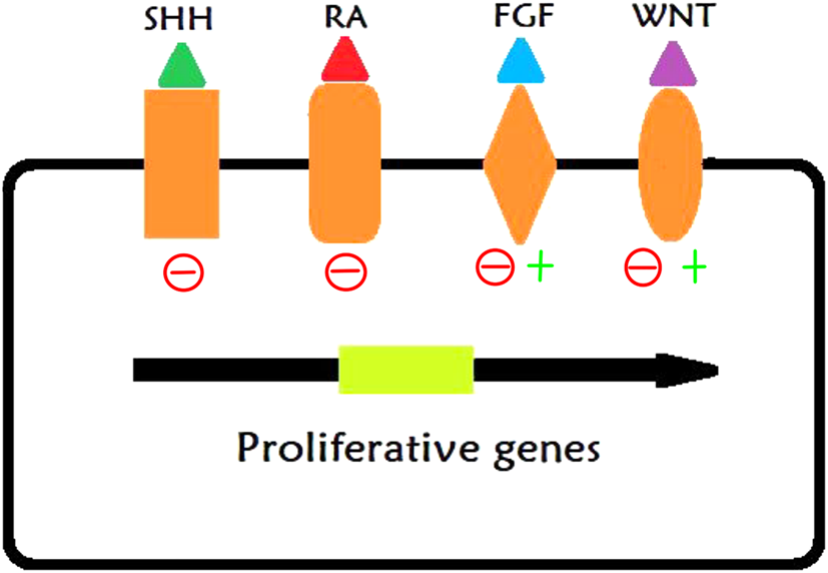
Effects of different pathways on the proliferation genes of progenitors. During the development of progenitor cells, the switch from differentiation to proliferation is regulated by specific factors (such as SHH, RA, WNT, FGFs)

Understanding the complex and plastic brain neural circuitry is a significant challenge. In particular, understanding cell migration and transformation in the context of brain development has been the goal of many researchers. Because lineage tracing can target specific progenitors and their progeny, it has been used to investigate the processes regulating brain development. Neural progenitor cells give rise to neurons, astrocytes and oligodendrocytes in the neocortex [30] (Fig. 2). During telencephalon development, neural progenitor cells predominantly differentiate into cortical and subcortical neurons. In the later stages of development, the progenitors give rise to stem cells, glial cells and ependymal cells. The various glial cells produced by neural precursor cells have different destinations in the developing brain (Fig. 2). Tracing studies show that the multipotency of neural progenitors and NSCs is regulated by Sonic hedgehog signaling [31, 32] (Fig. 3). Using genetic and pharmacological tools to measure and manipulate Wnt/β-catenin signaling in active and quiescent adult NSCs, both in vivo and in vitro, Oberst identified Wnt signaling as a core molecular pathway in the differentiation of apical progenitors originating from the subventricular zone (SVZ) [33]. Their study demonstrated plasticity in certain stages of neurogenesis. By crossing Grem1creERT; Rosa26LSLTdtomato mice and Emx1-cre-mediated Grem1 conditional knockout mice, Ichinose revealed that the bone morphogenetic protein (BMP) antagonist Grem1 promotes structural and functional maturation of the developing cortex [34]. Furthermore, orthodenticle homeobox 1 (Otx1) was identified as a key element in cortical neurogenesis. To examine the mechanism by which Otx1 affects cortical neurogenesis, Huang performed lineage tracing on Otx1 knockout mice and found that loss of function of Otx1 results in the overproduction of astrocytes in vivo [35].

Diverse neurons in the brain can also be traced to their source by lineage tracing. Neurogliaform cells, which have highly distinctive shapes and properties, act as inhibitory interneurons in the cerebral cortex. Inhibitory interneurons play an important role in maintaining the function of cortical microcircuits. Through in vivo genetic lineage tracing in mice, we demonstrated that neurogliaform cells are a distinct class of interneurons with a unique developmental trajectory [36]. While lineage tracing has been successfully used to identify the origin of interneurons in the cerebral cortex, the origin of hippocampal interneurons remains controversial [37]. Asgarian and colleagues used genetic lineage tracing and single-cell transcriptomic analysis to elucidate the origin of hippocampal CA1 somatostatin-expressing interneurons in mice [38]. These investigators found that functional heterogeneity within somatostatin CA1-expressing interneurons was not attributable to differences in origin, because all hippocampal CA1 somatostatin interneurons arise from the embryonic medial ganglionic eminence/preoptic area (MGE/POA). Compared with the hippocampus, there are relatively few studies on dentate gyrus (DG) development. Because of the advantages of lineage tracing, it has also been used investigate DG development. Lineage tracing has shown that intermediate progenitors (Tbr2^+^) interact, support and guide migrating NSCs (SOX9^+^), which generate all prenatal and postnatal granule neurons in a specific spatiotemporal order [39].

Astrocytes, which are the most abundant glial cells in the brain, exhibit local heterogeneity in morphology, gene expression and function. Olig2-lineage mature astrocytes are found in the forebrain of adult transgenic mice, and Tatsumi et al. demonstrated that these astrocytes differ from glial fibrillary acidic protein (GFAP)-positive astrocytes in distribution pattern, and that they may be involved in inhibitory neuronal transmission [40]. Clavreul et al. explored the mechanisms underlying astrocyte heterogeneity using the Brainbow lineage tracing technique, involving “large-volume color imaging”, and found that the final characteristics of astrocytes are determined by their interactions with neighboring cells and the microenvironment [41].

Although our understanding of the mechanisms underlying the formation of neocortical neurons and astrocytes has improved, less is known about oligodendrocytes. Lineage tracing also significantly increases the visibility of oligodendrocytes. Winkler et al. used genetic lineage tracing to clarify the origin and differentiation of dorsal forebrain oligodendrocyte progenitors, and demonstrated that most oligodendrocytes are derived from Emx1^+^ dorsal forebrain progenitors in the embryonic neocortex [42]. Moreover, they showed that Sonic hedgehog is necessary for oligodendrocyte generation, and that oligodendrocytes in specific germinal zones play a role in cell heterogeneity. Dorsally derived oligodendrocyte precursor cells continuously expand throughout the life of mammals, whereas ventrally derived oligodendrocyte precursor cells gradually diminish in number [43]. In the adult brain, neural and oligodendrocyte precursor cells in the SVZ contribute to oligodendrogenesis throughout life. By infusing fractalkine into the lateral ventricle of neural precursor cells in adult mice, Watson and colleagues demonstrated that fractalkine signaling plays an important regulatory role in oligodendrogenesis [44].

## Lineage Tracing for Analyzing Neurogenesis in the Adult Central Nervous System (CNS)

Neurogenesis has been identified in certain regions of the adult brain, in which NSCs can generate new neurons and glial cells throughout the life of mammals (including in the neurogenic niches of the hippocampal DG and ventricular SVZ) [45, 46]. Adult neurogenesis is a form of plasticity that persist through the life and was often implicated in recovery from injury. Because of the intricacies of the brain and the heterogeneity of NSCs, technical advances are needed for a more detailed understanding of neurogenesis in the brain. Lineage tracing has been widely applied to investigate neurogenesis in the brain, and has revealed the complex heterogeneity of NSCs within the SVZ, which is the main anatomical area in which NSCs are found [47]. Using retroviral lineage tracing in triple transgenic mice, Sachewsky et al. demonstrated that Oct4-expressing NSCs are precursors of GFAP-expressing NSCs in vivo [48]. ScRNA-seq has been used to examine the heterogeneity of NSCs, and has improved the resolution of lineage tracing. Using high-resolution scRNA-seq, Xie identified various subpopulations of NSCs in distinct regions of the SVZ [49]. Additionally, single-cell optical phenotyping and a new sequencing method combining live-cell imaging and scRNA-seq have enabled study of the cellular dynamics of neurogenesis in the SVZ of the adult brain, revealing substantial heterogeneity. It has been shown that Notum negatively regulates ventricular-subventricular zone (V-SVZ) proliferation [50]. However, the maintenance and generation of the NSC pool remain poorly understood. For example, the adult ventricular zone contains quiescent GFAP^+^ cells that have neurogenic potential in vivo, but contribute little to the activated NSC pool under both basal and regenerating states. This suggests that cells in the adult V-SVZ niche follow distinct neurogenic pathways [51]. By combining short- and long-term lineage tracing methods, Obernier et al. revealed that the NSC pool in the adult mouse V-SVZ is primarily maintained via symmetric divisions [52]. A recent study of transgenic mice expressing a fluorescent marker driven by the vascular endothelial-cadherin promoter suggested that progenitor pools originate not only from symmetric divisions, but also from endothelial cells [53]. NSCs in the SVZ have also been traced using the UbC-StarTrack clonal methodology by Figueres-Onate et al., who demonstrated that neural progenitor cells in the SVZ give rise to glial cells in the ventricular zone and adjacent areas, and to interneurons distributed throughout the olfactory bulb [47]. The vast majority of postnatal- and adult-born interneurons in the olfactory bulb are inhibitory. However, in a study using NeuroD6CreERT2 knockin mice and Rosa26tdTomato reporter mice, excitatory glutamatergic neurons were observed during certain periods in adulthood [54]. By combining long-term lineage tracing assays using two knockin alleles and quantitative clonal analysis, studies have suggested that the fate and number of NSCs are determined by a niche-based mechanism [55, 56].

Adult mouse hippocampal NSCs generate new neurons that are integrated into existing hippocampal networks to modulate mood and memory, and the proportion of quiescent NSCs increases with age [45, 57, 58]. The majority of NSCs within the brain remain in a reversible quiescent state outside of the cell cycle [59]. In the brain, Tweety-homolog 1 (Ttyh1) is highly expressed in NSCs and precursor cells. Using a Ttyh1 promoter-driven reporter and Ttyh1 knockout mice, Cao et al. showed that Ttyh1 inhibits the transition of NSCs from a quiescent to an activated state [60]. Moreover, RNA-seq, bioinformatics and molecular biological analyses have demonstrated that Ttyh1 regulates NSCs via calcium signaling. However, current models of the life cycle of hippocampal stem cells remain controversial because of their heterogeneity [61]. Increasing evidence from lineage tracing studies has demonstrated that NSCs in the hippocampus are heterogeneous populations with distinct markers, such as SOX2, NES and GFAP. The heterogeneity of NSCs in the hippocampus has been identified by scRNA-seq analysis of the GLI family zinc finger 1 and Achaete-scute homolog 1 lineages [62]. Recently, it was revealed that vascular cell adhesion molecule 1, a cell surface marker, is expressed in a subpopulation of NSCs in the adult mouse hippocampus. Dan-Ying and colleagues performed lineage tracing of vascular cell adhesion molecule 1-positive cells, and showed that they are quiescent and capable of generating neurons and astrocytes [63]. Therefore, these cells show promise as markers for differentiating between quiescent and active NSCs. Lineage tracing has also revealed SOX1-expressing NSC/progenitor cell populations in the hippocampus, which give rise to granular neurons and astrocytes and decrease in size with aging.

A niche-based regulatory mechanism of the fate of NSCs has also been demonstrated in the adult hippocampus using lineage tracing. Microglia modulate the balance between proliferation and survival in the neurogenic niche via their secretome, thereby supporting long-term adult hippocampal neurogenesis [64–66]. By combining optogenetics and lineage tracing technology, recent studies have shown that, in adults, DG parvalbumin-positive interneurons control whether NSCs remain quiescent or are activated [46, 67]. The Wnt/β-catenin signaling pathway plays an important regulatory role in the hippocampal neurogenic lineage. Through scRNA-seq of quiescent and active hippocampal NSCs in vivo, Austin et al. showed that both cell types respond to Wnt/β-catenin signaling in a dose-dependent manner [68]. Cannabinoid type-1 receptors expressed in NSCs and their progeny were identified as crucial regulators of the communication between the extracellular and cellular compartments [69].

## Lineage Tracing in CNS Diseases

The wide variety of cells and the complexity of neural networks renders the diagnosis and treatment of CNS diseases difficult. Lineage tracing is an effective method for monitoring cells of interest (and their progeny) over the long term, and has been used to explore CNS diseases. To examine how early damage can lead to disease in later life, Mohammad et al. generated transgenic reporter mouse lines as a novel tool for long-term, systemic tracking of cells (and their progeny) that sustained damage in the prenatal environmental [70]. They demonstrated that the long-term effects of prenatal exposure to environmental insult are mediated by altered regulation of key molecules, which can cause epigenetic modifications that may be inherited by progeny. The fate of cells after CNS diseases, such as multiple sclerosis and intracerebral hemorrhage, has also been elucidated by lineage tracing and single-cell sequencing in various diseases models [71, 72]. Recent studies suggest that Irx3 and Irx5, in association with intronic variants of fat mass and obesity-associated gene, are determinants of obesity. Moreover, scRNA-seq using the Ins2-Cre system revealed a previously unreported radial glial cell-like NSC population with high Irx3 and Irx5 expression, and demonstrated that Irx3 and Irx5 are critical regulators of feeding and leptin responses [73]. Functional recovery following brain injury has long been a research focus. Lineage tracing has been applied to investigate the response of SVZ NSCs to stroke. In a photothrombotic stroke model, lineage tracing revealed that nestin-positive endogenous NSCs originating from the SVZ show increased proliferation in response to stroke, migrate toward the infarct region, and differentiate into astrocytes and neurons. Moreover, these processes are exacerbated by excessive use of the limbs [74, 75]. However, lineage tracing studies of the response of stem cells in the SVZ to traumatic brain injury have yielded inconsistent results. The migration and differentiation of NSCs from the SVZ are hindered by gliosis, but can be improved by reducing glial scar formation [76]. Fibrous scars caused by brain injury have also been tracked by lineage tracing, which has revealed therapeutic targets [77].

## Lineage Reprogramming in the Brain

NSCs and progenitor cells have long been considered a promising resource for the repair of brain injury in adults; however, their potential for repairing extensive and chronic lesions is limited. To overcome this limitation, a lineage reprogramming approach has been developed to promote cellular regeneration. Several research groups have attempted to convert resident cells within damaged tissue into desired cell types via in vivo lineage reprogramming and tracing. Xiang conducted a lineage tracing study that targeted astrocytes, and provided strong evidence that astrocytes targeted by Aldh1-1 can be directly converted into neurons following adeno-associated virus-mediated expression of NeuroD1 [78]. However, the catalyst for the transformation in vivo remains unclear, despite the use of sophisticated lineage tracing techniques [79, 80].

During mouse corticogenesis, progenitor cells in the cerebral cortex behave in accordance with their intrinsic characteristics and environmental niches, and recent studies show that Otx1 plays an important role in the regulation of homeobox-containing transcription factors in the CNS. In a study of cortical progenitor cells and their progeny in Otxl-knockdown mice, the loss of Otx1, a key regulator of cortical neurogenesis, appeared to contribute to the overproduction of astrocytes in vivo [35]. During hippocampal neurogenesis, NSCs in the hippocampus can develop into neurons and astrocytes, but not into oligodendrocytes. Harris demonstrated that Nfix is necessary for neuroblast maturation and survival, and that NSCs can develop into oligodendrocytes following induced ablation of Nfix, as demonstrated by lineage tracing, transcriptomic sequencing and behavioral studies [81]. In addition, injecting SOX2, a transcription factor, into the adult mouse brain causes the conversion of astrocytes into neurons. To reverse the demyelination and associated secondary axonal damage seen in multiple sclerosis, Farhangi and colleagues transduced astrocytes with lentiviral vectors expressing SOX2-green fluorescent protein, and demonstrated that SOX2 can convert astrocytes into oligodendrocyte progenitor cells in mice exhibiting demyelination [82]. Moreover, SOX2 is required for the neurogenic reprogramming of oligodendrocyte precursor cells during recovery from spinal cord injury [83]. There is also evidence that astrocytes can be generated from oligodendrocyte precursor cells. Hou generated two lines of oligodendrocyte lineage-specific mice, and demonstrated, using lineage tracing, that conditional inactivation of Pen-2 leads to an increase in the number of astrocytes without any change in the number of neurons in the CNS [84].

## Conclusion

The CNS is the most complex organ in the body. Its numerous cell types and intricate connections pose challenges to our understanding of the CNS, including the complex processes of brain development in the embryonic period, as well as neurogenesis in the mature CNS and the changes that brain cells undergo during pathological conditions. The application of lineage tracing has greatly improved our understanding of complex brain mechanisms, including changes during disease processes. To elucidate the complicated transition of neural progenitors during the embryonic period, lineage tracing has been widely used to investigate the destination of neural progenitor cells through the combined use of different genetic markers. Lineage tracing has demonstrated that neural progenitors can differentiate into neurons, glia and neural stem cells in a specific spatiotemporal order. The production of neurons in the lower cortex is significantly higher than in the upper cortex. The progeny of neural progenitors are highly heterogeneous, as revealed by lineage tracing. By combining target knockout with inhibitor treatment, lineage tracing can also be used to investigate the regulation of neural progenitor development by different signaling pathways during embryonic development and neurogenesis in the adult. The elaboration of regulatory pathways has deepened our understanding of neural network development and promoted the application of pluripotent cells in diseases. Many patients with stroke or trauma show functional recovery of damaged brain areas after injury; however, the source of the neurons involved in the repair process remains unclear. Lineage tracing can be used to provide insight into the cell and molecular mechanisms of compensatory neural processes in different diseases, as well as clarify disease pathogenesis. Lineage tracing can also be used in the modulation of cell differentiation. By reprogramming stem cells, they can differentiate into desired cell types. Not only can they be reprogrammed into neurons, but also glial cells, providing the basis for post-injury brain network reconstruction. Despite the advantages of lineage tracing, its application to the brain also has some limitations. For example, lineage tracing models usually rely on tamoxifen induction. However, recent scRNA-seq and adeno-associated virus studies showed that, in a tamoxifen-induced mouse model, tamoxifen and bromodeoxyuridine exert potent inhibitory effects on neurogenesis [85, 86]. Although the high resolution of scRNA-seq allows for accurate classification of cell types, individual cells cannot be tracked over space and time with such accuracy. Furthermore, complete lineage tracing in mammals remains challenging because of a lack of diversity of DNA barcodes. Most of the methods currently used primarily provide lineage relationship at a specific point in time. Therefore, for a more comprehensive appraisal of the developmental trajectories of glia and neurons, it is highly desirable to explore methods that reveal the relationship between different cells and their progeny. The combined application of multiple lineage tracing techniques should allow for in-depth analysis of the relationship between different cells. Moreover, the application of lineage tracing for the study of neurological diseases remains limited. Additional lineage tracing methods are required to address these issues and elucidate the complex pathological and physiological processes in the body, especially in the context of brain injuries. The advances made with these techniques should encourage clinical translation.

## Data Availability

Not applicable.

## Abbreviations

BMP: Bone morphogenetic protein
CNS: Central nervous system
Cre: Cyclization recombination enzyme
CRISPR/Cas: Clustered regularly interspaced short palindromic repeat/associated nuclease
DG: Dentate gyrus
Dre: D6 site-specific DNA recombinase
FGF: Fibroblast growth factor
FLP-FRT: Flippase–flippase recognition target
GFAP: Glial fibrillary acidic protein
MGE/POA: Medial ganglionic eminences/preoptic area
NSCs: Neural stem cells
Otx1: Orthodenticle homeobox 1
RA: Retinoic acid
ScRNA-seq: Single-cell RNA-sequencing
SHH: Signaling molecule sonic hedgehog
SVZ: Subventricular zone
Ttyh1: Tweety-homolog 1

## References

[1] Triarhou LC, Manto M (2022). Postnatal neurogenesis beyond rodents: The groundbreaking research of Joseph Altman and Gopal Das. Cerebellum.

[2] Ming G-L, Song H (2011). Adult neurogenesis in the mammalian brain: Significant answers and significant questions. Neuron.

[3] Marques S, van Bruggen D, Vanichkina DP, Floriddia EM, Munguba H, Varemo L (2018). Transcriptional convergence of oligodendrocyte lineage progenitors during development. Developmental Cell.

[4] Zhang Y, Zeng F, Han X, Weng J, Gao Y (2020). Lineage tracing: Technology tool for exploring the development, regeneration, and disease of the digestive system. Stem Cell Research & Therapy.

[5] Kretzschmar K, Watt FM (2012). Lineage tracing. Cell.

[6] Wu Y, Han X, Su Y, Glidewell M, Daniels JS, Liu J (2021). Multiview confocal super-resolution microscopy. Nature.

[7] Weisblat DA, Sawyer RT, Stent GS (1978). Cell lineage analysis by intracellular injection of a tracer enzyme. Science.

[8] Axelrod D (1979). Carbocyanine dye orientation in red cell membrane studied by microscopic fluorescence polarization. Biophysical Journal.

[9] Cotsarelis G, Sun TT, Lavker RM (1990). Label-retaining cells reside in the bulge area of pilosebaceous unit: Implications for follicular stem cells, hair cycle, and skin carcinogenesis. Cell.

[10] Fraint E, Ulloa BA, Feliz Norberto M, Potts KS, Bowman TV (2021). Advances in preclinical hematopoietic stem cell models and possible implications for improving therapeutic transplantation. Stem Cells Translational Medicine.

[11] Jin H, Liu K, Zhou B (2021). Dual recombinases-based genetic lineage tracing for stem cell research with enhanced precision. Science China Life Science.

[12] Price J, Turner D, Cepko C (1987). Lineage analysis in the vertebrate nervous system by retrovirus-mediated gene transfer. Proceedings of the National Academy of Sciences of the United States of America.

[13] Cepko C (1988). Retrovirus vectors and their applications in neurobiology. Neuron.

[14] Crowther AJ, Lim S-A, Asrican B, Albright BH, Wooten J, Yeh C-Y (2018). An adeno-associated virus-based toolkit for preferential targeting and manipulating quiescent neural stem cells in the adult hippocampus. Stem Cell Reports.

[15] Suzuki E, Nakayama M (2011). VCre/VloxP and SCre/SloxP: New site-specific recombination systems for genome engineering. Nucleic Acids Research.

[16] Nowak JA, Polak L, Pasolli HA, Fuchs E (2008). Hair follicle stem cells are specified and function in early skin morphogenesis. Cell Stem Cell.

[17] Madisen L, Zwingman TA, Sunkin SM, Oh SW, Zariwala HA, Gu H (2010). A robust and high-throughput Cre reporting and characterization system for the whole mouse brain. Nature Neuroscience..

[18] Chen M-R, Liu S-W, Wu T-C, Kao VY, Yu H-C, Chen FH (2010). RU486-inducible recombination in the salivary glands of lactoferrin promoter-driven green fluorescent cre transgenic mice. Genesis.

[19] Poulin JF, Luppi MP, Hofer C, Caronia G, Hsu PK, Chan CS (2020). PRISM: A progenitor-restricted intersectional fate mapping approach redefines forebrain lineages. Developmental Cell..

[20] Gao P, Postiglione MP, Krieger TG, Hernandez L, Wang C, Han Z (2014). Deterministic progenitor behavior and unitary production of neurons in the neocortex. Cell.

[21] Livet J, Weissman TA, Kang H, Draft RW, Lu J, Bennis RA (2007). Transgenic strategies for combinatorial expression of fluorescent proteins in the nervous system. Nature.

[22] Zhou F, Li X, Wang W, Zhu P, Zhou J, He W (2016). Tracing haematopoietic stem cell formation at single-cell resolution. Nature.

[23] Zafar H, Lin C, Bar-Joseph Z (2020). Single-cell lineage tracing by integrating CRISPR-Cas9 mutations with transcriptomic data. Nature Communications.

[24] Pandey S, Shekhar K, Regev A, Schier AF (2018). Comprehensive Identification and spatial mapping of habenular neuronal types using single-cell RNA-seq. Current Biology.

[25] Raj B, Wagner DE, McKenna A, Pandey S, Klein AM, Shendure J (2018). Simultaneous single-cell profiling of lineages and cell types in the vertebrate brain. Nature Biotechnology.

[26] Wagner DE, Weinreb C, Collins ZM, Briggs JA, Megason SG, Klein AM (2018). Single-cell mapping of gene expression landscapes and lineage in the zebrafish embryo. Science.

[27] Yao M, Ren T, Pan Y, Xue X, Li R, Zhang L (2022). A new generation of lineage tracing dynamically records cell fate choices. International Journal Molecule Science.

[28] Pei W, Wang X, Rössler J, Feyerabend TB, Höfer T, Rodewald H-R (2019). Using Cre-recombinase-driven Polylox barcoding for in vivo fate mapping in mice. Nature Protocols.

[29] Bowling S, Sritharan D, Osorio FG, Nguyen M, Cheung P, Rodriguez-Fraticelli A (2020). An Engineered CRISPR-Cas9 mouse line for simultaneous readout of lineage histories and gene expression profiles in single cells. Cell.

[30] Ojalvo-Sanz AC, Lopez-Mascaraque L (2021). Gliogenic potential of single pallial radial glial cells in lower cortical layers. Cells.

[31] Hashimoto Y, Gotoh H, Ono K, Nomura T (2019). Differential potentials of neural progenitors for the generation of neurons and non-neuronal cells in the developing amniote brain. Scientific Reports.

[32] Zhang Y, Liu G, Guo T, Liang XG, Du H, Yang L (2020). Cortical neural stem cell lineage progression is regulated by extrinsic signaling molecule sonic hedgehog. Cell Reports.

[33] Oberst P, Fievre S, Baumann N, Concetti C, Bartolini G, Jabaudon D (2019). Temporal plasticity of apical progenitors in the developing mouse neocortex. Nature.

[34] Ichinose M, Suzuki N, Wang T, Kobayashi H, Vrbanac L, Ng JQ (2021). The BMP antagonist gremlin 1 contributes to the development of cortical excitatory neurons, motor balance and fear responses. Development.

[35] Huang B, Li X, Tu X, Zhao W, Zhu D, Feng Y (2018). OTX1 regulates cell cycle progression of neural progenitors in the developing cerebral cortex. Journal of Biological Chemistry.

[36] Niquille M, Limoni G, Markopoulos F, Cadilhac C, Prados J, Holtmaat A (2018). Neurogliaform cortical interneurons derive from cells in the preoptic area. eLife.

[37] Chittajallu R, Craig MT, McFarland A, Yuan X, Gerfen S, Tricoire L (2013). Dual origins of functionally distinct O-LM interneurons revealed by differential 5-HT(3A)R expression. Nature Neuroscience.

[38] Asgarian Z, Magno L, Ktena N, Harris KD, Kessaris N (2019). Hippocampal CA1 somatostatin interneurons originate in the embryonic MGE/POA. Stem Cell Reports.

[39] Nelson BR, Hodge RD, Daza RA, Tripathi PP, Arnold SJ, Millen KJ (2020). Intermediate progenitors support migration of neural stem cells into dentate gyrus outer neurogenic niches. eLife.

[40] Tatsumi K, Isonishi A, Yamasaki M, Kawabe Y, Morita-Takemura S, Nakahara K (2018). Olig2-lineage astrocytes: A distinct subtype of astrocytes that differs from GFAP astrocytes. Frontiers in Neuroanatomy.

[41] Clavreul S, Abdeladim L, Hernandez-Garzon E, Niculescu D, Durand J, Ieng SH (2019). Cortical astrocytes develop in a plastic manner at both clonal and cellular levels. Nature Communications.

[42] Winkler CC, Yabut OR, Fregoso SP, Gomez HG, Dwyer BE, Pleasure SJ (2018). The dorsal wave of neocortical oligodendrogenesis begins embryonically and requires multiple sources of sonic hedgehog. Journal of Neuroscience.

[43] Liu R, Jia Y, Guo P, Jiang W, Bai R, Liu C (2021). In vivo clonal analysis reveals development heterogeneity of oligodendrocyte precursor cells derived from distinct germinal zones. Advance Science.

[44] Watson AES, de Almeida MMA, Dittmann NL, Li Y, Torabi P, Footz T (2021). Fractalkine signaling regulates oligodendroglial cell genesis from SVZ precursor cells. Stem Cell Reports.

[45] Moreno-Jimenez EP, Terreros-Roncal J, Flor-Garcia M, Rabano A, Llorens-Martin M (2021). Evidences for adult hippocampal neurogenesis in humans. Journal of Neuroscience.

[46] Matsubara S, Matsuda T, Nakashima K (2021). Regulation of adult mammalian neural stem cells and neurogenesis by cell extrinsic and intrinsic factors. Cells.

[47] Figueres-Onate M, Sanchez-Villalon M, Sanchez-Gonzalez R, Lopez-Mascaraque L (2019). Lineage tracing and cell potential of postnatal single progenitor cells in vivo. Stem Cell Reports.

[48] Sachewsky N, Xu W, Fuehrmann T, van der Kooy D, Morshead CM (2019). Lineage tracing reveals the hierarchical relationship between neural stem cell populations in the mouse forebrain. Scientific Reports.

[49] Xie XP, Laks DR, Sun D, Poran A, Laughney AM, Wang Z (2020). High-resolution mouse subventricular zone stem-cell niche transcriptome reveals features of lineage, anatomy, and aging. Proceeding of National Academy Science USA.

[50] Mizrak D, Bayin NS, Yuan J, Liu Z, Suciu RM, Niphakis MJ (2020). Single-cell profiling and SCOPE-seq reveal lineage dynamics of adult ventricular-subventricular zone neurogenesis and NOTUM as a key regulator. Cell Reports.

[51] Joppe SE, Cochard LM, Levros LC, Hamilton LK, Ameslon P, Aumont A (2020). Genetic targeting of neurogenic precursors in the adult forebrain ventricular epithelium. Life Science Alliance.

[52] Obernier K, Cebrian-Silla A, Thomson M, Parraguez JI, Anderson R, Guinto C (2018). Adult neurogenesis is sustained by symmetric self-renewal and differentiation. Cell Stem Cell.

[53] Soto-Avellaneda A, Morrison BE (2020). Central nervous system and peripheral cell labeling by vascular endothelial cadherin-driven lineage tracing in adult mice. Neural Regeneration Research.

[54] Angelova A, Platel JC, Beclin C, Cremer H, Core N (2019). Characterization of perinatally born glutamatergic neurons of the mouse olfactory bulb based on NeuroD6 expression reveals their resistance to sensory deprivation. The Journal of Comparative Neurology.

[55] Basak O, Krieger TG, Muraro MJ, Wiebrands K, Stange DE, Frias-Aldeguer J (2018). Troy+ brain stem cells cycle through quiescence and regulate their number by sensing niche occupancy. Proceeding of National Academy Science USA.

[56] Borrett MJ, Innes BT, Jeong D, Tahmasian N, Storer MA, Bader GD (2020). Single-cell profiling shows murine forebrain neural stem cells reacquire a developmental state when activated for adult neurogenesis. Cell Reports.

[57] Kalamakis G, Brune D, Ravichandran S, Bolz J, Fan W, Ziebell F (2019). Quiescence modulates stem cell maintenance and regenerative capacity in the aging brain. Cell.

[58] Pilz G-A, Bottes S, Betizeau M, Jörg DJ, Carta S, Simons BD (2018). Live imaging of neurogenesis in the adult mouse hippocampus. Science.

[59] Urbán N, van den Berg DLC, Forget A, Andersen J, Demmers JAA, Hunt C (2016). Return to quiescence of mouse neural stem cells by degradation of a proactivation protein. Science.

[60] Cao Y, Wu HN, Cao XL, Yue KY, Han WJ, Cao ZP (2021). Transmembrane protein Ttyh1 maintains the quiescence of neural stem cells through Ca(2+)/NFATc3 signaling. Frontier Cell Development Biology.

[61] Lazutkin A, Podgorny O, Enikolopov G (2019). Modes of division and differentiation of neural stem cells. Behavior Brain Research.

[62] Bottes S, Jaeger BN, Pilz G-A, Jörg DJ, Cole JD, Kruse M (2021). Long-term self-renewing stem cells in the adult mouse hippocampus identified by intravital imaging. Nature Neuroscience..

[63] Dan-Ying W, An-Feng W, Bai LQ-R, Gong X-L, Zheng Y, Shen Q (2020). VCAM1 labels a subpopulation of neural stem cells in the adult hippocampus and contributes to spatial memory. Stem Cell Reports.

[64] Choe Y, Pleasure SJ, Mira H (2015). Control of adult neurogenesis by short-range morphogenic-signaling molecules. Cold Spring Harbor Perspectives Biology.

[65] Diaz-Aparicio I, Paris I, Sierra-Torre V, Plaza-Zabala A, Rodríguez-Iglesias N, Márquez-Ropero M (2020). Microglia actively remodel adult hippocampal neurogenesis through the phagocytosis secretome. Journal of Neuroscience.

[66] Harley SBR, Willis EF, Shaikh SN, Blackmore DG, Sah P, Ruitenberg MJ (2021). Selective ablation of BDNF from microglia reveals novel roles in self-renewal and hippocampal neurogenesis. Journal of Neuroscience.

[67] Song J, Zhong C, Bonaguidi MA, Sun GJ, Hsu D, Gu Y (2012). Neuronal circuitry mechanism regulating adult quiescent neural stem-cell fate decision. Nature.

[68] Austin SHL, Gabarro-Solanas R, Rigo P, Paun O, Harris L, Guillemot F (2021). Wnt/beta-catenin signalling is dispensable for adult neural stem cell homeostasis and activation. Development.

[69] Zimmermann T, Maroso M, Beer A, Baddenhausen S, Ludewig S, Fan W (2018). Neural stem cell lineage-specific cannabinoid type-1 receptor regulates neurogenesis and plasticity in the adult mouse hippocampus. Cerebral Cortex..

[70] Mohammad S, Page SJ, Sasaki T, Ayvazian N, Rakic P, Kawasawa YI (2020). Long-term spatial tracking of cells affected by environmental insults. Journal of Neurodevelopmental Disorder.

[71] Dorrier CE, Aran D, Haenelt EA, Sheehy RN, Hoi KK, Pintaric L (2021). CNS fibroblasts form a fibrotic scar in response to immune cell infiltration. Nature Neuroscience.

[72] Shi SX, Shi K, Liu Q (2021). Brain injury instructs bone marrow cellular lineage destination to reduce neuroinflammation. Science Translational Medicine.

[73] Son JE, Dou Z, Kim KH, Wanggou S, Cha VSB, Mo R (2021). Irx3 and Irx5 in Ins2-Cre(+) cells regulate hypothalamic postnatal neurogenesis and leptin response. Nature Metabolism.

[74] Vandeputte C, Reumers V, Aelvoet SA, Thiry I, De Swaef S, Van den Haute C (2014). Bioluminescence imaging of stroke-induced endogenous neural stem cell response. Neurobiology of Diseases.

[75] Liang H, Zhao H, Gleichman A, Machnicki M, Telang S, Tang S (2019). Region-specific and activity-dependent regulation of SVZ neurogenesis and recovery after stroke. Proceedings of the National Academy of Sciences of the USA.

[76] Wang Z, Zheng Y, Zheng M, Zhong J, Ma F, Zhou B (2020). Neurogenic niche conversion strategy induces migration and functional neuronal differentiation of neural precursor cells following brain injury. Stem Cells Development.

[77] Dias DO, Kalkitsas J, Kelahmetoglu Y, Estrada CP, Tatarishvili J, Holl D (2021). Pericyte-derived fibrotic scarring is conserved across diverse central nervous system lesions. Nature Communications..

[78] Xiang Z, Xu L, Liu M, Wang Q, Li W, Lei W (2021). Lineage tracing of direct astrocyte-to-neuron conversion in the mouse cortex. Neural Regeneration Research.

[79] Zhang Y, Li B, Cananzi S, Han C, Wang LL, Zou Y (2022). A single factor elicits multilineage reprogramming of astrocytes in the adult mouse striatum. Proceedings of the National Academy of Sciences of the USA.

[80] Wang LL, Serrano C, Zhong X, Ma S, Zou Y, Zhang CL (2021). Revisiting astrocyte to neuron conversion with lineage tracing in vivo. Cell.

[81] Harris L, Zalucki O, Clement O, Fraser J, Matuzelski E, Oishi S (2018). Neurogenic differentiation by hippocampal neural stem and progenitor cells is biased by NFIX expression. Development.

[82] Farhangi S, Dehghan S, Totonchi M, Javan M (2019). In vivo conversion of astrocytes to oligodendrocyte lineage cells in adult mice demyelinated brains by Sox2. Multiple Sclerosis Relate Disorder.

[83] Tai W, Wu W, Wang LL, Ni H, Chen C, Yang J (2021). In vivo reprogramming of NG2 glia enables adult neurogenesis and functional recovery following spinal cord injury. Cell Stem Cell.

[84] Hou J, Bi H, Ye Z, Huang W, Zou G, Zou X (2021). Pen-2 negatively regulates the differentiation of oligodendrocyte precursor cells into astrocytes in the central nervous system. Journal of Neuroscience.

[85] Lee C-M, Zhou L, Liu J, Shi J, Geng Y, Liu M (2020). Single-cell RNA-seq analysis revealed long-lasting adverse effects of tamoxifen on neurogenesis in prenatal and adult brains. Proceedings of the National Academy of Sciences of the USA.

[86] Wang T, Liao J-C, Wang X, Wang Q-S, Wan K-Y, Yang Y-Y (2022). Unexpected BrdU inhibition on astrocyte-to-neuron conversion. Neural Regeneration Research..

[87] Chen C, Liao Y, Peng G (2022). Connecting past and present: Single-cell lineage tracing. Protein & Cell.

